# Cigarette smoke-induced chronic obstructive pulmonary disease is attenuated by CCL20-blocker: a rat model

**DOI:** 10.3325/cmj.2016.57.363

**Published:** 2016-08

**Authors:** Desheng Sun, Yao Ouyang, Yanhui Gu, Xiansheng Liu

**Affiliations:** 1 Department of Respiratory and Critical Care Medicine, Tongji Hospital, Tongji Medical College, Huazhong University of Science and Technology, Wuhan, China; 2 Department of Respiratory Medicine, Affiliated Hospital of Zunyi Medical College, Zunyi, China

## Abstract

**Aim:**

To evaluate whether the effect of dendritic cells (DCs) on chronic obstructive pulmonary disease (COPD) can be relieved by blocking CCL20.

**Methods:**

30 Wistar rats were randomly divided into three groups: control, COPD, and COPD treated with CCL20 monoclonal antibody. In the latter two groups, COPD was induced by four-week cigarette smoke exposure and trachea injection of lipopolysaccharide solution on two occasions. CCL20 monoclonal antibody was injected intraperitoneally on the first day. All animals were sacrificed on the 29th day. Pathomorphology of the lung and bronchiole was analyzed using hematoxylin and eosin staining. The CCR6 content in the bronchoalveolar lavage fluid was detected using ELISA. DC distribution in the lung was examined by immunohistochemistry for OX62.

**Results:**

COPD rat models showed pathological alterations similar to those in COPD patients. DCs, CCR6, and the severity of emphysema were significantly increased in the COPD group than in controls (all *P* values <0.001), and they were significantly reduced after anti-CCL20 treatment compared with the COPD group (all *P* values <0.05).

**Conclusion:**

The interaction between CCR6 and its ligand CCL20 promotes the effect of DCs in the COPD pathogenesis, which can be reduced by blocking CCL20.

Cigarette smoking is the primary risk factor for the development of chronic obstructive pulmonary disease (COPD), the fourth most common cause of death worldwide (1,2). The characteristic irreversible airflow limitation of COPD and its increasing prevalence worldwide (3) have stimulated much research into its pathogenesis. This disease is associated with a specific inflammatory response of the small airways, which causes small airway obstruction and lung parenchyma destruction. Studies have demonstrated that immune system cells play an important role in the inflammation response, however, the precise mechanisms leading to this type of inflammation remain unknown. The organization of lymphocytes into lymphoid follicles and the presence of oligoclonal lymphocytes suggest that immune responses in COPD are at least partly driven by specific antigens (4-6).

Such immune responses are under control of dendritic cells (DCs) (7,8). Airway DCs initiate and regulate adaptive immune responses in the lung (8). They form a highly sensitive sentinel network around the airways, and are able to migrate through the intact epithelium to sample foreign antigens within the airway lumen (9). After antigen uptake, DCs migrate to the draining lymph nodes to convey antigenic information to specialized lymphocytes, which organize an inflammatory response against the encountered antigen (8). Thus, the whole process of DC migration can be divided into two steps: antigen capture and antigen presentation (10). There is growing evidence that the information required for the regulation of leukocyte traffic is provided by the differential distribution of chemokines, together with the flexible usage of chemokine receptors (11). During the first step, an important role is played by several chemokines and chemokine receptors. The major chemokine that attracts DCs is CCL20, which has been proposed to induce LC recruitment at sites of inflammation (12). The only receptor for CCL20 is CCR6 (13), expressed on immature DCs (14).

DCs play a crucial part in the pathogenesis of airway inflammation in asthma (15,16). However, there is limited information on the role of DC and its chemokines in COPD. Our previous studies have shown that DCs are accumulated and CCR6/CCL20 levels are increased in the airways of patients with COPD (17,18), which corresponds with findings of other studies (19,20). However, the precise mechanism is still unclear. Therefore, we aimed to determine whether the effect of DCs on COPD can be relieved by blocking CCL20.

## Materials and methods

### Animals

Homozygous male Wistar WT rats (8 weeks old) were obtained from the experimental animal center of the Third Military Medical University (Chongqing, China). 30 rats used for this experiment were divided into three groups by a random number table method: COPD model, control, and CCL20 monoclonal antibody treated (MAT) group, each consisting of 10 rats. The animal experiments were carried out in accordance with the recommendations from the Guide for the Care and Use of Laboratory Animals of the National Institutes of Health (21), and all *in vivo* experiments were approved by the Animal Experimentation Ethics Committee of the Zunyi Medical College (Guizhou Province, China).

### Experimental design

The rat model of COPD was established by smoke exposure and intratracheal instillation of lipopolysaccharide (LPS) as described previously (22,23). Experimental rats, including the COPD group and the CCL20 monoclonal antibody treated group, underwent whole body exposure to tobacco smoke of 12 cigarettes in a tobacco smoke chamber (90 cm ×40 cm ×30 cm, made of Plexiglas) designed by one of the authors (YG) twice a day with 2-hour smoke-free intervals, every day for four weeks. The smoke exposure experimental box consists of a box body and a cover, with a smoke inlet at the bottom part of the box body and a smoke outlet on the side wall of the top part. The smoke-to-air ratio was 1:7, to protect the rats from acute smoke toxicity and death. The controls were exposed to air.

Animals from the two smoke-exposed groups were administered 200 µg/200 µL of LPS solution intratracheally on two occasions, on the first and the 15th day of tobacco smoke exposure. Additionally, rats from the CCL20 monoclonal antibody treated group were injected intraperitoneally with CCL20 monoclonal antibody (100 μg per rat, our preliminary experimental results showed this dose contributed to an ideal effect) on the first day of the experiment. All rats were sacrificed on the 29th day. These experiments were repeated three times.

### Bronchoalveolar lavage (BAL)

Rats were weighed 24 hours after the last smoke exposure and sacrificed with an overdose of pentobarbital, and a tracheal cannula was inserted. 1 mL of Hank's balanced salt solution (HBSS), free of ionized calcium and magnesium but supplemented with 0.05 mM sodium EDTA (Sigma, St Louis, MO, USA), was instilled four times via the tracheal cannula and recovered by gentle manual aspiration. The four lavage fractions were centrifuged, the cell pellet was washed twice, and resuspended in 1 mL of HBSS.

### Histology and morphometric analysis

The left lung was fixated by gentle infusion of fixative (4% paraformaldehyde) through the tracheal cannula. After excision, the lung was immersed in fresh fixative for 2 h. A lung lobe was embedded in paraffin and cut in 3-µm transversal sections. Lung tissue samples were stained with hematoxylin and eosin (HE) and examined by light microscopy for histological sections. For each animal, 10 fields at a magnification of 100 × were captured randomly from 4 different zones of the left lung (upper, middle upper, middle basal, and basal zone) by laboratory technicians using a IPWin32 image analyzer platform (Leica, Wetzlar, Germany). The mean alveolar number (MAN) was obtained and proportion of alveolar area (PAA) was measured using Image-Pro Plus 6.0 software (Media Cybernetics, Rockville, MD, USA).

### Measurement of chemokine receptors

Using a commercially available enzyme-linked immunosorbent assay (ELISA) kit (West Tang Biotech, Shanghai, China), CCR6 protein level was determined in BAL fluid (BALF). BALF samples were centrifuged at 1500 rpm for 10 min, and the supernatant was collected for further analysis. The detection threshold for this system was 15 ng/L. The main process of ELISA method was as follows: We allowed all reagents to reach room temperature before use, and then gently mixed all liquid reagents before use. We then set up blank wells (blank control without samples and HRP-conjugate reagent, and the rest of the steps were the same). We added 100 µL of prepared standard and sample to wells, covered the plate, and incubated it at room temperature for 2 hours. Solution from wells was thoroughly aspirated, and the liquid was discarded, after which the wells were washed 4 times using an automated 96-well plate washer. We added 100 µL of diluted detection antibody to wells, covered the plate, and incubated it at room temperature for 1 hour. Solution from the wells was thoroughly aspirated, the liquid discarded, and the wells were washed 4 times. 100 µL of diluted HRP-conjugate was added to each well and cover plate and incubated at room temperature for 30 minutes. The solution from wells was thoroughly aspirated, the liquid discarded, and the wells were washed 4 times. 100 µL of chromogenic substrate was added to each well, followed by 100 µL of stop solution. The solution in the wells should have changed from blue to yellow, and the plate had to be evaluated within 30 minutes of stopping the reaction. A blank well was taken as zero adjustment, and the absorbance of each well was read at 450 nm. Curve-fitting statistical software was used to plot a four-parameter logistic curve fit to the standards and the results were then calculated for the test samples.

### DC study by immunohistochemistry

Serial sections obtained from formalin-fixed, paraffin-embedded lung lobes were stained by immunohistochemistry with an antibody against the DC marker OX62 (CD103) using a modified protocol. In brief, sections were deparaffinized, rehydrated, and submerged in methanol 10% H_2_O_2_ to block endogenous peroxidase activity. The sections were then washed with phosphate buffered saline (PBS) and a microwave antigen retrieval was performed (middle fire, 10 min). Then, sections were washed with PBS and incubated (10 min) with universal blocking solution (Laboratory Vision Corp, Fremont, CA, USA) and normal goat serum (30 min) to block any nonspecific antibody binding. Subsequently, sections were incubated with the mouse anti-rat OX62 (1/100) antibody (Thermo Fisher Scientific Inc, Waltham, MA, USA) overnight in a humidity chamber at 4°C. They were washed in PBS and treated (30 min) with EnVisionTM Detection Kit (the secondary antibody system produced by Gene Tech, Shanghai, China). Afterward, they were washed in PBS and visualized using the EnVisionTM DAB+ Chromogen stained for 7 min for the anti-OX62 immunostaining, counterstained with hematoxylin and mounted in an aqueous medium. As negative controls, other sections were incubated with PBS instead of the primary antibody. The morphology and distribution of stained cells were analyzed under light microscopy. Integral optical density (IOD) of the positive areas was analyzed using IPWIN60 software (Media Cybernetics, Inc, Rockville, MD, USA) and the results were used as semiquantitative DC amounts.

### Statistical analysis

Data are presented as mean ± standard deviation. Statistical analyses were performed with SPSS software (version 17.0; SPSS Inc., Chicago, IL, USA). All data were tested for normality of distribution using Shapiro-Wilk test. Because the data were normally distributed, a one-way analysis of variance (ANOVA) was performed to compare means among the three groups. A value of *P* < 0.05 was considered significant.

## Results

### Pathological changes in the lungs

**Figure 1:**
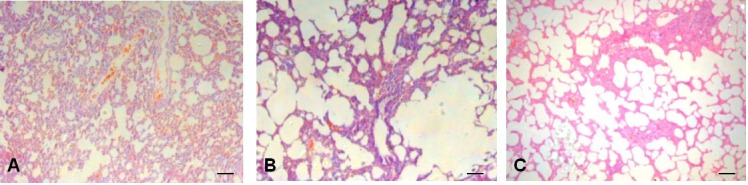
Photomicrographs of hematoxylin and eosin stained lung tissue of (**A**) controls, (**B**) chronic obstructive pulmonary disease models, and (**C**) CCL20 monoclonal antibody treated group (original ×100, scale bar: 100 µm)

Rats in the COPD model group and CCL20 monoclonal antibody treated group showed lassitude, loss of hair luster, and less activity. Microscopic analysis of lung tissue sections revealed a considerable degree of chronic bronchitis and emphysema, and clearly induced alveolar wall destruction (Figure 1). HE staining showed that the pathological changes in the COPD group were similar to those of COPD patients (24,25). In controls there was no inflammatory cell infiltration and the gland in the small bronchus, the alveolar wall was intact, and the alveolar structure was complete and continuous (Figure 1A). In the COPD group, alveolar intervals were widened, with lymphocytes, mononuclear cells, and neutrophil infiltration. Capillary dilation and congestion were found in the intervals, and partial alveolar wall destruction was clearly induced (Figure 1B). In the CCL20 monoclonal antibody treated group, the alveolar septa were widened, accompanied by inflammatory cell infiltration and alveolar space expansion. However, the extent of the lesion was considerably reduced compared with the COPD group (Figure 1C). MAN was significantly lower in the COPD group than in controls (283.0 ± 26.52 vs 68.23 ± 20.74; *P* < 0.001) and significantly higher in the CCL20 monoclonal antibody treated group than in the COPD group (115.8 ± 14.44 vs 68.23 ± 20.74; *P* = 0.021). PAA was significantly greater in COPD group than in the control group (76.64 ± 4.179 vs 39.63 ± 2.848; *P* < 0.001) and significantly smaller in the CCL20 monoclonal antibody treated group than in the COPD group (62.39 ± 3.422 vs 76.64 ± 4.179; *P* = 0.010). (Table 1)

**Table 1:** Morphologic indices of pulmonary emphysema in controls, rats with chronic obstructive pulmonary disease (COPD), and rats treated with CCL20 monoclonal antibody (MAT) (mean ± standard deviation)

Group	Mean alveolar number ( × 10^6^/m^2^)	Proportion of alveolar area (%)
Controls	283.0 ± 26.52	39.63 ± 2.848
COPD group	68.23 ± 20.74*	76.64 ± 4.179*
CCL20 MAT group	115.8 ± 14.44^†^	62.39 ± 3.422^‡^

*P < .001 vs controls.

†*P* = 0.021 vs COPD group.

‡*P* = 0.010 vs COPD group.

### DC morphology and distribution

**Figure 2:**
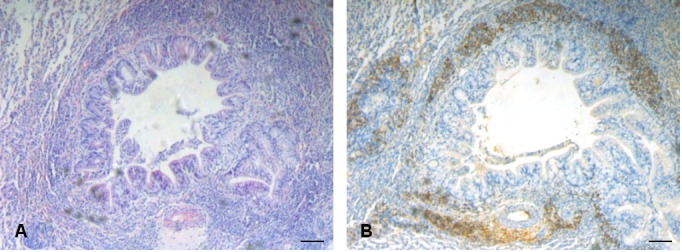
(**A**) Photomicrograph of hematoxylin and eosin stained tissue. **(B**) Dendritic cells identified as brown-stained cells by using immunohistochemistry for OX62 expression in paraffin-embedded rat lung sections (chronic obstructive pulmonary disease model group, original ×100, scale bar: 120 µm).

**Figure 3:**
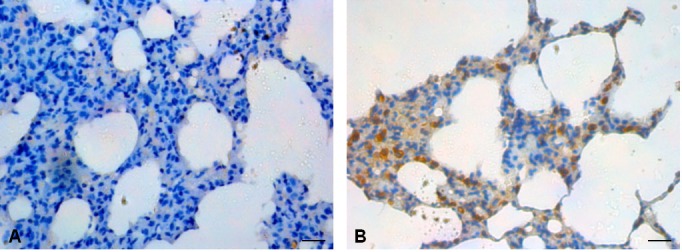
(**A**) A negative control (sections incubated with phosphate buffered saline instead of the primary antibody). (**B**) Dendritic cells identified as brown-stained cells by using immunohistochemistry for OX62 expression (immunopositive cells were stained as brown in their cytoplasm) (chronic obstructive pulmonary disease group, original ×400, scale bar: 30 µm).

OX62+ cells were located mainly in the subepithelial areas of the airway wall, distributed between the lamina propria and adventitia, especially congregated around the air passage (Figure 2). Positive cells were stained as brown in their cytoplasm (Figure 3).

### Immunohistochemistry study for OX62

**Figure 4:**
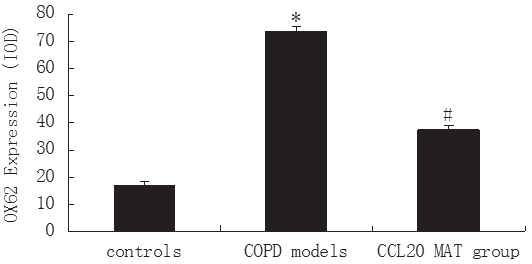
OX62 expression in the lung tissue of controls, chronic obstructive pulmonary disease group, and CCL20 monoclonal antibody treated group (n = 10 per group). Values in black bars are mean ± standard deviation. *P < 0.001 vs controls, #*P* < 0.001 vs chronic obstructive pulmonary disease group.

**Figure 5:**
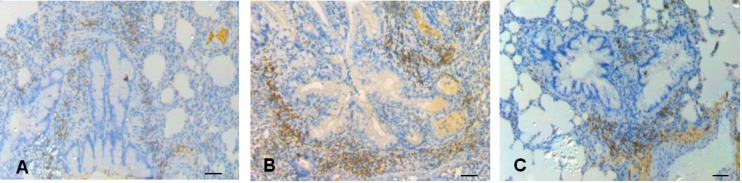
Photomicrographs of immunohistochemistry study for OX62 expression in (**A**) controls, (**B**) chronic obstructive pulmonary disease group, and (**C**) CCL20 monoclonal antibody treated group (original ×200, scale bar: 50 µm).

The number of IOD-comprising OX62+ cells was significantly increased in the COPD group compared with controls (17.07 ± 1.35 vs 73.45 ± 2.21; *P* < 0.001), and was significantly decreased in the CCL20 monoclonal antibody treated group compared with the COPD group (37.58 ± 1.55 vs 73.45 ± 2.21; *P* < 0.001) (Figure 4, Figure 5).

### CCR6 levels in the BAL fluid

**Figure 6:**
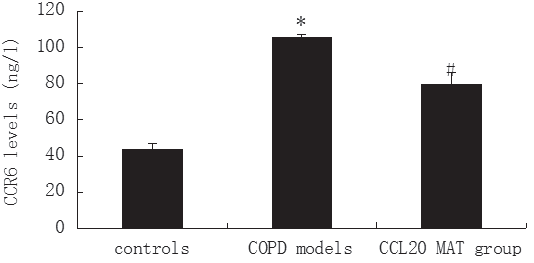
CCR6 levels in the bronchoalveolar lavage fluid of controls, chronic obstructive pulmonary disease (COPD) group, and CCL20 monoclonal antibody treated group (n = 10 per group). Values in black bars are mean ± standard deviation. *P < 0.001 vs controls, #*P* < 0.001 vs COPD group.

The content of CCR6 in BALF was significantly increased in the COPD group compared with controls (105.81 ± 1.58 ng/L vs 43.57 ± 3.18 ng/L; *P* < 0.001) and significantly decreased in the CCL20 monoclonal antibody treated group compared with the COPD group (79.84 ± 6.49 ng/L vs 105.81 ± 1.58 ng/L; *P* < 0.001) (Figure 6).

## Discussion

Our study showed that COPD symptoms and pathomorphology were relieved in rats injected intraperitoneally with CCL20 monoclonal antibody, and that DC number and CCR6 level were lower than in COPD rats. This might be due to the lack of CCL20, combined with the CCL20 monoclonal antibody.

Smoke-induced alterations to pulmonary DCs lead to the development of inappropriate immunological responses in susceptible smokers and an abnormal inflammatory reaction to antigens, favoring the development of COPD (26). Infection is also an important factor in the COPD pathogenesis (27), with considerable prevalence among COPD patients (28). Thus, we established a rat COPD model by cigarette smoke exposure and LPS solution injection through the trachea, which could imitate the COPD pathogenesis in humans better than exposure to cigarette smoke or LPS injection alone (29). Since smoking and infection are the two most important environmental factors that cause the COPD development, our method is more similar to the natural process of COPD pathogenesis. Furthermore, this model is more stable and less time-consuming (30).

Few studies have investigated the role of DCs in the COPD pathogenesis. Some animal models (20,31-33) of emphysema showed an increase in DC number, while others showed a decrease. Patients with COPD were shown to have significantly increased small airway langerin+ DCs (19). We showed that OX62+ DCs number in the lungs and CCR6 levels were elevated in rats with COPD. However, these parameters were decreased in the CCL20 monoclonal antibody treated group compared with the COPD group. OX62 (also known as CD103), first found in the lymphoid tissue of rats, has been recognized as a specific marker for rat DC (34,35).

The elevated number of pulmonary DCs following COPD occurrence suggests a possible role for these cells in the COPD pathogenesis. Clearly, DCs play a major role in the innate immune response and are the most powerful antigen-presenting cells in the respiratory tract. The DC network within and under the epithelium of the conducting airways is ideally positioned to perform a sentinel role against harmful inhalants. The traffic of DCs is facilitated by a sequence of chemotactic stimuli, and the expression of corresponding chemokine receptors on DCs (10). Our study also indicates that CCR6/CCL20 played a critical role in DC accumulation into epithelial tissues when COPD occurred.

A limitation of the study may be the absence of a control group with an already used treatment in COPD rat model, but this was not of the utmost importance for the purposes of this study. Also, further investigation into the intracellular molecular regulating mechanism is needed.

In summary, using a rat COPD model we demonstrated that CCL20 blocking impaired DCs accumulation in the rat lung. This may in part indicate that the interaction of CCR6 with its ligand CCL20 contributes to the COPD pathogenesis. In addition, CCL20 monoclonal antibody might provide a possible treatment option for patients with COPD. However, the selected dose of the antibody is different in different species (36), which is why further studies are needed to determine its therapeutic effect and optimal dosage in humans.
